# A Pilot Study on Falling-Risk Detection Method Based on Postural Perturbation Evoked Potential Features

**DOI:** 10.3390/s19245554

**Published:** 2019-12-16

**Authors:** Shenglong Jiang, Hongzhi Qi, Jie Zhang, Shufeng Zhang, Rui Xu, Yuan Liu, Lin Meng, Dong Ming

**Affiliations:** 1 School of Precision Instrument & Opto-Electronics Engineering, Tianjin University, Tianjin 300072, China; justinjiang@tju.edu.cn (S.J.); jie_zhangtju@163.com (J.Z.); shufeng1994@tju.edu.cn (S.Z.); xrblue@tju.edu.cn (R.X.); 2 Academy of Medical Engineering and Translational Medicine, Tianjin University, Tianjin 300072, China; ryanliu@tju.edu.cn (Y.L.); linmeng@tju.edu.cn (L.M.)

**Keywords:** brain–computer interface (BCI), electroencephalogram (EEG), cross-task recognition, falling-risk detection, postural perturbation, machine learning

## Abstract

In the human-robot hybrid system, due to the error recognition of the pattern recognition system, the robot may perform erroneous motor execution, which may lead to falling-risk. While, the human can clearly detect the existence of errors, which is manifested in the central nervous activity characteristics. To date, the majority of studies on falling-risk detection have focused primarily on computer vision and physical signals. There are no reports of falling-risk detection methods based on neural activity. In this study, we propose a novel method to monitor multi erroneous motion events using electroencephalogram (EEG) features. There were 15 subjects who participated in this study, who kept standing with an upper limb supported posture and received an unpredictable postural perturbation. EEG signal analysis revealed a high negative peak with a maximum averaged amplitude of −14.75 ± 5.99 μV, occurring at 62 ms after postural perturbation. The xDAWN algorithm was used to reduce the high-dimension of EEG signal features. And, Bayesian linear discriminant analysis (BLDA) was used to train a classifier. The detection rate of the falling-risk onset is 98.67%. And the detection latency is 334ms, when we set detection rate beyond 90% as the standard of dangerous event onset. Further analysis showed that the falling-risk detection method based on postural perturbation evoked potential features has a good generalization ability. The model based on typical event data achieved 94.2% detection rate for unlearned atypical perturbation events. This study demonstrated the feasibility of using neural response to detect dangerous fall events.

## 1. Introduction

In the human-robot hybrid system, it is hard to avoid error recognition, which may lead to poor interaction experience and falling-risk by erroneous motor execution, which may lead to falling-risk and is hardly detected by itself. However, the cerebral cortex plays an import role in the control of cognitive function and behavior control in human-environment interaction. At present, the detection performance of human beings to complex dangerous events is incomparable to those of mostly artificial intelligence systems. Combining advantages of human nervous system can effectively improve the security of human-machine interaction system. The current brain-computer interface (BCI) system mainly focuses on improving recognition precision of operation intention, but only few studies on the neural mechanism of erroneous motion status detection, and no application based on this kind of neural response.

Unpredictable postural perturbation is a common dangerous situation that changed the original dynamic balanced state of the body and could lead to a fall. Since walk path and speed are variant toward changing of target position and obstacle, the timing and force of postural perturbation are always unexpected. Thus, postural perturbation caused falling-risk cannot be ignored for elderly persons, especially the motor dysfunctional patients with hemiplegic or paralysis. The last decade has seen a tremendous amount of researches in the field of falling-risk detection, which mainly based on kinematic sensors and computer vision [1,2]. The kinematic sensors and computer vision on fall detection with motion recognition always behind a fall. Noury et al. reviewed kinematics fall detection method from impact shock of acceleration and velocity vector and suggested that such methods result in high false positive rates [3]. Alzahrani et al. used fixed Kinect V2 camera to capture human body motion and trained a fuzzy classifier by 3D skeleton features, which results in more than 94.5% accuracy of fall frame [4]. However, the human central neural system controls complex sensorimotor function and regulates interactions for motor planning, execution, and sensor feedback [5,6]. The human sensorimotor system shows remarkable skills in perceiving subtle balance changes [7].

The monitoring of motor execution requires the integration of sensory information from visual, vestibular, and proprioceptive somatosensory inputs [8,9]. Recent researches proposed a cortical response mechanism of postural control focused on perturbation-evoked potentials [10] and functional near-infrared spectroscopy (fNIRS) [11]. Moreover, EEG has highly temporal precision to detect time-locked event-related cortical activity. Previous studies revealed that perturbation events evoked profound negativity within 100ms (N1 potentials) [12,13]. A previous study reported a cortical theta power increase in frontal and central areas, primarily located along the midline cortical, which is correlated with sudden postural perturbation [14]. Other studies suggested that theta band activity in frontal and central cortical areas are involved in the monitoring of postural stability and dangerous events during balance control [15,16]. Although it has been found that postural perturbation can lead to changes in neurophysiological signals of the brain, there is no research attempting to use brain signals as a biomarker to detect it. We have reason to believe that EEG biomarker could be a new detection method of falling-risk in a human-machine interactive system.

Typically, falling is a high-risk event for motor dysfunctional patients. The spinal cord injuries (SCI) lead to damage in various degrees of motor and sensory function [17]. And, the gait feature of SCI individuals depends on damage degree, recovery stage, and individual characteristics. Meanwhile, the falling-risk onset is a complex and varying situation. Fall events occur infrequently and collecting fall data in motor dysfunctional patients may be harmful, which leads to numeric and species short of the dataset for training classifiers. Therefore, the need for a generic falling-risk detection method in motor dysfunctional patients is much stronger than healthy people in rehabilitation treatment, as well as daily life.

In this paper, we aim to evaluate the detection performance using dynamic neural responses during different types of falling-risk, which included the detection rate, detection latency when in instability state, and false alarm rate in stability state. And we attempted to establish a generic model for cross-task detection based on neural response features of ipsilateral postural perturbation events, which can also detect contralateral perturbation events. We expect that BCI system could be a viable application of the general dangerous events detection method helpful to motor dysfunctional patients.

## 2. Materials and Methods

### 2.1. Subjects and Experimental Paradigm

In this study, we simulated two types of falling-risk events on the subjects without movement intention which caused by unpredictably random erroneous motion execution of the external equipment. We tested the neural and behavioral responses of those rapidly unpredictable postural perturbation events. 15 healthy subjects participated in the experiment (23-28 years, body mass 50-75 kg, all right-handedness, 6 males and 9 females). The study was approved by the ethical committee of Tianjin University (Tianjin, China). All subjects signed informed consent in advance.

**Figure 1:**
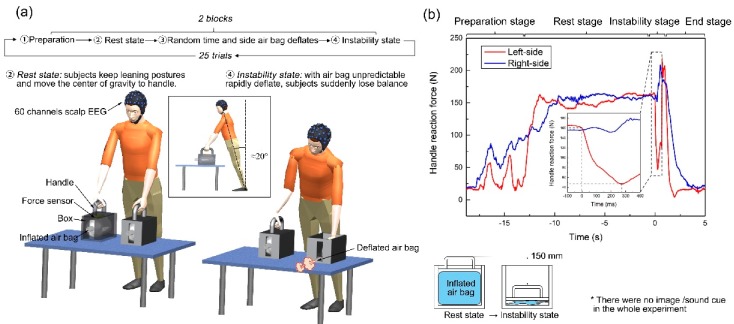
(**a**) Experimental paradigm and equipment. (**b**) Typical force in Newtons applied on an assistance handle overtime during a balance perturbation experiment run. Force was measured using a sensor on the handle. In the preparation stage, the subjects placed their hands on handles and leaned forward, gradually moving their center of gravity towards the handles. In the instability stage, unpredictable airbag exhaust breaks the original body balance of subjects, subjects responded to the postural perturbation and try to restore balance. In the end, the subjects relaxed and returned to natural upright standing.

Figure 1a showed the experimental paradigm and equipment in this study. The experiment equipment consisted of an inflatable airbag that could exhaust rapidly, a handle that moved up-down when the airbag inflated and deflated, a force sensor connected to both the handle and the airbag, and also a box used to contain the devices described above. The participants remain on the rest state for a random period between 5 to 15 s and then suddenly switch to the instability state by random side of airbag deflation. During the rest state, the airbag was fully inflated, and the subjects kept a stable standing while leaning forward approximately 20 degrees, and moved the center of gravity towards the handles (simulating walker-assisted standing of SCI individuals). In the instability state, one of the two handles (randomly selected) will rapidly move down, simulating falling due to deflation of the airbag (which takes maximum to 300 ms). The subject lost balance as the support force of one of the upper limbs decreases rapidly. The experiment performs 50 events per subject.

Figure 1b shows the applied force in Newton on the left handle overtime during a typical balance perturbation. Notice on the top of the figure the time sections for preparation, rest, instability, and end are clearly marked. During the instability stage, the force applied to the handle decreased rapidly more than 100 N within 300 ms. While decreasing of unilateral handle reaction force, the force of contralateral limb alternative increased to prevent falling. During the experiment, postural perturbation caused a noticeable behavioral response: when subjects felt imbalance at the beginning of instability state, they tried to shift their center of gravity to the opposite upper limb to prevent falling. We marked the starting point of the instability state according to the maximum second derivative value of the transient handle reaction force; this starting point is also used in the analysis synchronous evoked neural and behavioral responses.

### 2.2. Data Recording

EEG activity from 60 scalp channels was recorded via a Neuroscan SynAmps2 EEG amplifier (Compumedics Neuroscan, Australia). All the electrodes were placed based on the international 10–10 system. The reference electrode and ground electrode were placed at central region and AFz channel. The impedance of the electrodes was kept below 5 KΩ. The raw EEG and force signals were acquired at a sampling rate of 1000 Hz and a 0 to 200 Hz bandpass filter were applied to EEG recordings. The force sensors are metal strain gauge-based, they provide reaction force direction, full-scale capacity of 400 N, accurate to 0.02%. EEG and handle force were simultaneously recorded.

### 2.3. Removal of Potential Interfering Factors

In order to avoid EEG interference from motion artifacts, event expectation, or sound stimulation, we applied the following measures. For motion artifact caused by the movement of the electrode during the experiment, a bandage was used to fix the EEG cap, and the 0.1–10 Hz bandpass filter removed the EMG artifacts in EEG (main frequency is 500–2000 Hz) and partly unnecessary EEG components of the most sensorimotor rhythm for avoiding the interference of active motion intention by event related (de)synchronization (ERD/ERS) phenomenon [18] and slow cortical potential (SCP) [19,20]. For avoiding event expectation effects, the postural perturbation events happened at an unpredictable timing and side (left or right) without any image or sound cue. At the meanwhile, to reduce auditory evoked potential, the airbag exhaust noise was silenced. The averaged ambient noise level measured at the head position of subjects was 55 dB. When the airbag deflated, the noise level was less than 65 dB.

### 2.4. EEG Feature Extraction and Pattern Recognition

The raw EEG signals were bandpass filtered by Butterworth filter of 0.1-10 Hz and down-sampled to 50 Hz (5 times of the effective frequency band, preserving the original characteristics of the signal while improving the processing speed). The continuous EEG was segmented into epochs from t = −2 to 4 s relative to the perturbation event onset. We stochastically split the 50 trials into training and testing datasets by 45, and 5 with no trial mixture in between datasets. The temporal epochs length of train set input to classifier was chosen to be typically 500 ms. In addition, data augmentation of train set with each trials was performed by randomly selecting 20 rest state epochs and sequentially selected 20 instability state epochs using sliding from [−300 ms, 200 ms] to [100 ms, 600 ms] by 20 ms per step. In ttest set, we sequentially extracte epochs from −500 ms to 500 ms by sliding with 20 ms per step, for testing performance of classifier around perturbation event, and performed 10-fold cross-validation to test the robustness of the classification performance.

The xDAWN spatial filtering algorithm was chosen to enhance the signal-to-noise ratio of postural perturbation evoked potentials, generating a time-locked one-dimensional evoked synchronous response [21]. The Z-score normalization was applied to the output filters. In order to ensure the reliability of the test and more close to the actual use, the filter and normalization parameters applied to the test set were obtained from train set.

The classifier used in this work was Bayesian linear discriminant analysis (BLDA), which used regularization to avoid over-fitting to high dimensional and possibly noisy datasets [22]. The BLDA classifier threshold was set for each subject by the median of a train-set BLDA classifier output, to balance between sensitivity and specificity.

## 3. Results

### 3.1. Classification Performance of Postural Perturbation Event

**Figure 2:**
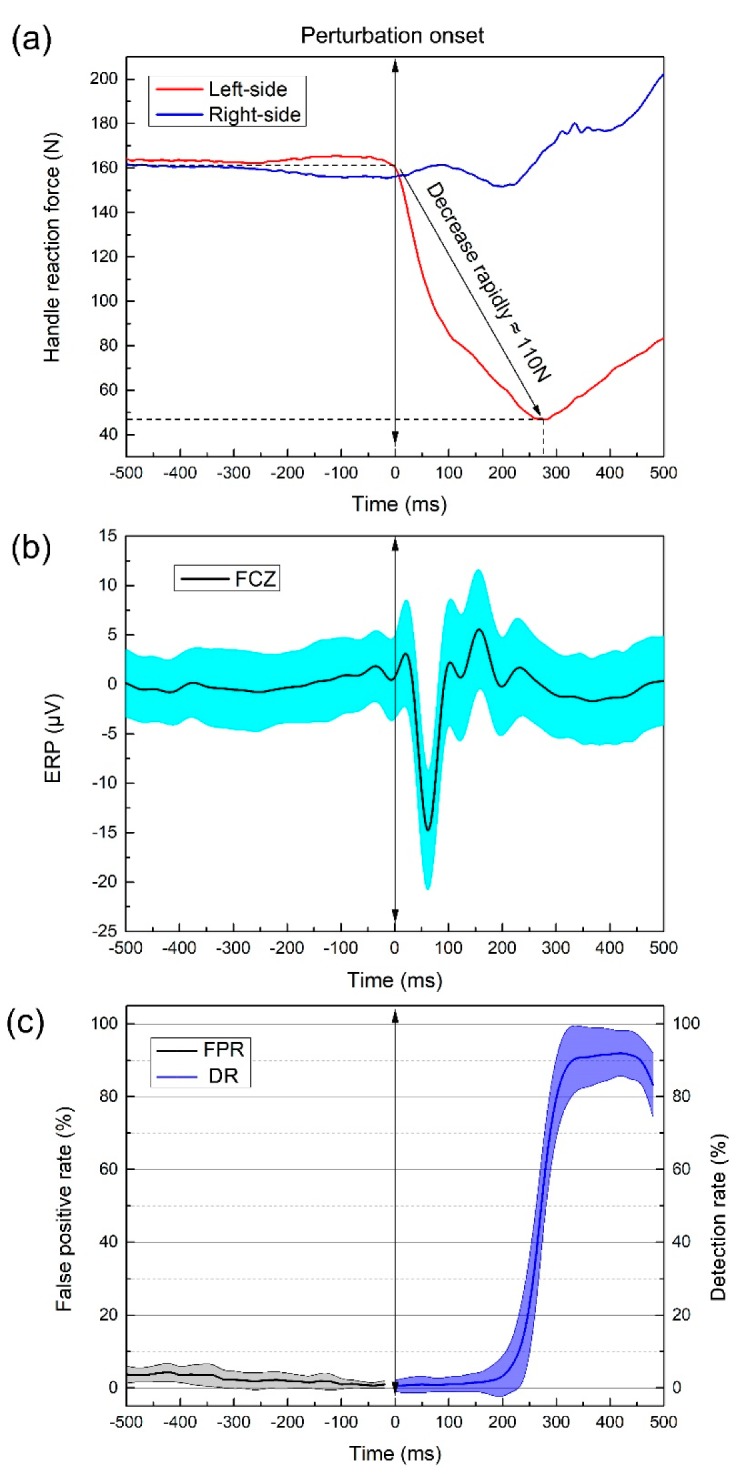
Synchronously (**a**) typical handle reaction force, (**b**) grand average FCZ channel ERP and (**c**) false alarm rate at rest state and detection rate at instability state for the falling-risk event.

For all subjects, a strong synchronous evoked neural responses and common behavioral responses were observed to be associated with the onset of postural perturbation. Figure 2 shows the time-synched handle reaction force, grand average event-related potential (ERP) at FCZ channel, and perturbation detection performance based on EEG features.

Figure 2a shows the handle reaction force during a typical left-side postural perturbation event. In the first 100 ms after the perturbation event, left-side handle reaction force rapidly decreased more than 80 N, and the maximum decline is about 130 N within 300 ms. On the contrary, the right-side handle reaction force increased. After that, both-side handle reaction force gradually increased, the human body completed the behavioral response to postural perturbation events.

Figure 2b shows baseline of potential at non-task state (−500 ms to 0) and the postural perturbation event evoked a nearly −15 μV negative potential at FCZ channel within 100 ms, which is much higher than most of sensory, visual, or auditory evoked potential. This phenomenon indicated that the human central nervous system had a rapid and strong response to postural perturbation events, which is consistent with numerous previous studies showing that perturbation evokes ERP at frontal-central channels [16,22] which constituted the foundation of perturbation detection based on EEG features.

Figure 2c shows the subject-specific perturbation detection results. The classifier kept a reliably high averaged detection rate up to 98.67%, while the classifier maintained an extremely low false alarm rate in rest state, the averaged false alarm rate of non-perturbation was 4.69%, to minimize the risk of fall by false triggering operation during the normal standing. During the first 300 ms, postural perturbation classifier was not very sensitive. However, the detection rate increased rapidly up to over 90% around 300 ms.

Table 1 shows detection performance by subject-specific classifier model for perturbation detection. Most subjects have an extremely low false alarm, three subjects’ averaged false alarm rates were less than 1%. If we set detection rate beyond 90% as the detection standard of perturbation event, the minimum detection latency is 278 ms and the maximum one is 334 ms. And, 11 subjects could reach the maximum detection rate of 100% in a short duration. Results in Table 1 show the feasibility of reliably fast perturbation detection method based on EEG feature, the maximum and minimum values were marked by bold.

### 3.2. Time-Spatial Distribution of ERP Component

**Figure 3:**
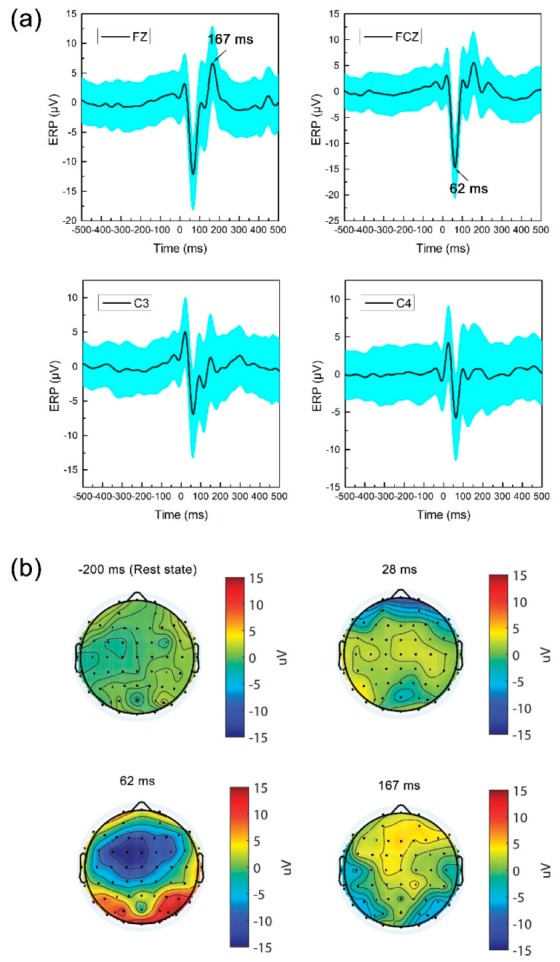
(**a**) Grand-average ERP record at midline channels, C3 and C4 channels; (**b**) Time-spatial topography in rest state and ERP peaking. The perturbation evoked negative potential was mainly located at frontal-central channels, especially mean amplitude peaking at FCZ around 62 ms.

To characterize perturbation evoked potentials, the latency and amplitude of ERP were measured for each subject. Figure 3 shows grand-average ERP within −500 to 500 ms and scalp topography at peaking. Falling-risk of postural perturbation as a dangerous condition, induced rapid responses of multiple brain regions to avoid falling. There was a clear negative peak amplitude of ERP following postural perturbation located at the frontal midline channel with a maximum averaged negative amplitude of −14.75 ± 5.99 μV at FCZ channel around 62 ms. As proposed in other studies, the current results present perturbation evoked ERP response that observed negativity potential within 100 ms (N1) following erroneous expectation [23]. In addition, there was an averaged positive amplitude of 5 μV at FZ channel around 167 ms. The perturbation evoked ERPs that have larger oscillation amplitudes than visual or auditory evoked potential, and the latency is very short. There was the foundation of the perturbation detection classifier.

### 3.3. Generalization Performance of Cross-Task Recognition

In the human walking process, there are many uncertain factors due to the varied walking path, target, and pavement condition. In reality, the sample size that can be collected is limited, and it is impossible to precisely model a realistic daily walking environment. Therefore, the generalization performance of the classifier is very important. Based on the same dataset, we restructured it to typical and atypical types, according to the perturbation event that happened on the left or right side. The evaluation method of generalization performance is atypical types of postural perturbation events recognition performance based on limited typical postural perturbation event dataset, which simulated the occurrence of unexpected and unknown perturbation events in reality. The results of the last section have shown that postural perturbation event induced significant neural responses, especially N1 potentials at FCZ channel.

**Figure 4:**
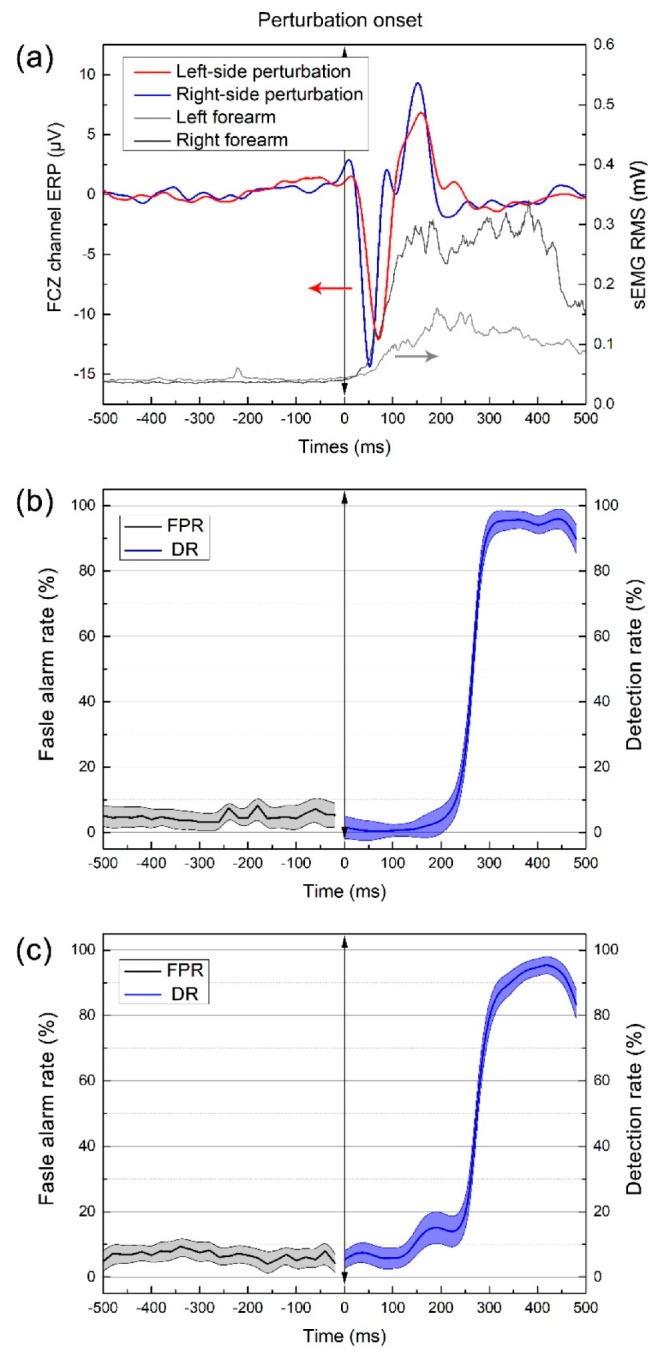
(**a**) Postural perturbation evoked ERP at FCZ channel and sEMG at wrist extensor during the right-side perturbation event. (**b**) Recognition performance of right-side postural perturbation event based on the left-side dataset; (**c**) Recognition performance of left-side postural perturbation event based on the right-side dataset.

As can be seen in Figure 4a, during left-side perturbation event, the human sensorimotor system rapidly detected the strong stimulus of postural perturbation and produced a behavioral response to restore body balance, this is shown in the different increased activation of both side wrist extensors; the muscle activation of the perturbation side increased much more than support side. Although the behavioral responses of different type perturbation events are significantly different, the perturbation evoked ERP of different types shows in Figure 4a are consistent.

In order to prove our hypothesis that postural perturbation evoked potential features have good cross-task recognition performance, we trained a classifier based on EEG features of a single type of typical postural perturbation events and tested its classification performance of unlearned different type of atypical postural perturbation. The results of Figure 4b and c are recognition performance for detecting unlearned right-side perturbation events based on EEG feature of left-side events and unlearned left-side perturbation events based on right-side events, respectively. The classifier had a low averaged false alarm rate of 4.7% and 8.6% at rest state, similar to the subject-specific model, and the highest detection rate of atypical perturbation reached 94.2%. This illustrates that the classifier established by typical dataset also had similar recognition capacity for unlearned atypical events. For patients, it is difficult to collect enough perturbation events through experiment or daily life, as well as cross-task detect unlearned atypical perturbation event which has a crucial impact on safety.

## 4. Discussion

The main purpose of this study was to propose a potential usage of EEG features in falling-risk detection. Based on classification performance and neural response results, the classifier based on perturbation evoked EEG features shows robust detection rate at instability state, few false alarm in rest state and short latency. The results show that stochastically unexpected postural perturbation evokes N1 potential at frontal-central channels, and these features could be used to detect the perturbation onset. And, the short latency of neural response feature allowed fast single trials identification of randomly postural perturbation.

There are two important differences between neural response and traditional kinematics based falling-risk detection methods. The first one is different types of postural perturbation can evoked potential in short latency, and neural response was always consistently sharing similar spatio-temporal characteristics [24,25]. Therefore, a generic falling-risk detection system could be created for different kinds of perturbation events without relying on a big dataset, sensor deployment, and working environment. The second one is that postural perturbation evoked potential amplitudes in unpredictable and predictable conditions are much different. Allan L et al. reported that unpredictable perturbations generated a large N1 potential, and that predictable perturbations instead produced an anticipation-related potential prior to the perturbation [26]. It showed that perturbation evoked potential features only evoked by unpredictable conditions, while normal motion execution did not generate similar ERP. Therefore, there is no conflict between normal active motion execution and erroneous motion status detection.

Cortical motor control is an interactive system of motor command and sensory feedback. A forward model in the sensorimotor system used to refine movement by comparing outgoing motor command and expected sensory consequence at a very short latency [27]. The sensorimotor system has an efficient error compensatory mechanism [28,29,30]. Previous studies had suggested that frontal-midline theta oscillation reflected a neural mechanism for human sensorimotor integration, which had previously been observed in recognition, attention tasks [23,27,31]. Coupling neural oscillations measured directly from human sensorimotor cortex by electrocorticogram (ECoG) also suggested theta rhythm activity was modulated by sensory, motor, and cognitive events [32]. Wessel et al. argued unexpected events interrupt action and impact cognition, partly at least by recruiting this global suppressive network. They described that a performance monitoring system serves to detect response-conflict of sensory response with motor execution, which produces N1 potential when there is a detected mismatch between a forward model and the motor efference copy [33,34]. Source localization study has shown that dipole locations of the N1 potential may lie in the supplementary motor area (SMA) [20], posterior cingulate motor area (pCMA) [35], and dorsal anterior cingulate cortex (dACC) [36]. In this study, the perturbation evoked potential features correlated with unexpected postural perturbation, which has a maximum amplitude of -15 μV within 100 ms, which is consistent with previous studies. Although the findings of these studies are generally consistent with the notion that brain structures are involved in perception and correction of postural perturbation, they require more research that combine the neural activities of afferent and efferent pathways. Also, certain kinds of feature selection methods may help reduce EEG channel numbers for improving device portability and processing speed [37].

The generalization performance of the classifier is an important issue in machine learning research. Due to the temporal and directional uncertainty of postural perturbation onset, falling-risk detection is a challenging topic. In particular, falling-risk events are dangerous and harmful to motor dysfunctional SCI individuals, which make it difficult to collect enough fall data for training the classifier in realism or simulations. Falling-risk events may induce a series of neural and behavioral responses, which come from the conditioned reflex accumulated in the process of biological evolution. In the face of danger, the human body will execute a series of movements to prevent falls, even with fear emotion and related facial expressions. It should be noted that the fall events are highly uncertain, thus corresponding limbs or neck muscle responses are also uncertain, which leads to the difficulty of signal acquisition and classifier training. Facial expression is dominated by the emotional response, which can’t precede the central nervous response in terms of latency. However, the emotional response evoked by the dangerous event, which is not easily separated from the EEG components, but this kind of evoked neural activity may be helpful to the classifier. In results, bilateral perturbation events evoked different behavioral characteristics in sEMG of both forearms, but the ERPs of FCZ channel are quite similar. Moreover, the classifier trained by dataset of single-type perturbation events, which could accurately detect the other type of perturbation events with high detection rates of 94.2% and low false positive rates of 4.7%. We revealed evidences of perturbation evoked potential would be a reliable feature for detecting different types of unpredictable falling-risk status. The generalization performance of the atypical falling-risk events recognition based on the perturbation evoked potential of limited dataset would be beneficial to fall detection in unlearned types of falling-risk events.

## 5. Conclusions

In conclusion, in this paper we demonstrated the potential application of a fast and reliable falling-risk postural perturbation detection by using perturbation evoked EEG features. The results showed a high detection rate of 98.67% with limited detection latency of 314.4 ms and a low false alarm rate of 2.56%. Moreover, we found that the classifier based on typical perturbation event evoked potential feature has an outstanding cross-task detection rate of up to 94.2% for unlearned atypical perturbation events. The generic ability of cross-task detection indicated the effectiveness and practicability of perturbation evoked potential features in falling-risk detection of erroneous motion status.

## Figures and Tables

**Table 1:** Falling-risk event detection performance of each subject.

Subject	Averaged False Alarm Rate	Detection Latency (Detection Rate Beyond 90%)	Maximum Detection Rate	Peak Timing
1	**0.32 ± 0.77%**	324 ± 26.33 ms	100.00%	348 ± 28.98 ms
2	8.24 ± 6.76%	326 ± 69.31 ms	**92.00%**	**362 ± 25.29 ms**
3	2.48 ± 2.15%	296 ± 30.98 ms	100.00%	320 ± 24.94 ms
4	0.48 ± 0.86%	312 ± 35.53 ms	94.00%	342 ± 23.94 ms
5	0.72 ± 2.01%	338 ± 28.98 ms	100.00%	356 ± 38.64 ms
6	4.16 ± 5.77%	**344 ± 39.78 ms**	100.00%	355 ± 34.06 ms
7	5.20 ± 6.57%	316 ± 15.78 ms	100.00%	322 ± 10.33 ms
8	4.72 ± 2.57%	334 ± 38.93 ms	100.00%	349 ± 15.78 ms
9	2.56±2.87%	316 ± 30.98 ms	96.00%	328 ± 30.07 ms
10	**13.04 ± 6.6%**	**278 ± 52.03 ms**	100.00%	308 ± 50.06 ms
11	3.76 ± 2.9%	282 ± 30.48 ms	100.00%	314 ± 38.85 ms
12	8.16 ± 4.97%	330 ± 38.01 ms	100.00%	336 ± 34.38 ms
13	6.64 ± 3.11%	320 ± 36.51 ms	100.00%	331 ± 18.85 ms
14	4.00 ± 2.75%	316 ± 29.51 ms	98.00%	319 ± 25.73 ms
15	2.48 ± 2.96%	284 ± 20.66 ms	**100.00%**	**304 ± 18.97 ms**
